# CDK11^p58^ inhibits ERα-positive breast cancer invasion by targeting integrin β3 via the repression of ERα signaling

**DOI:** 10.1186/1471-2407-14-577

**Published:** 2014-08-08

**Authors:** Yayun Chi, Sheng Huang, Lei Wang, Ruoji Zhou, Lisha Wang, Xiuying Xiao, Dali Li, Ying Cai, Xiaoyan Zhou, Jiong Wu

**Affiliations:** Breast Cancer Institute; Department of Breast Surgery, Fudan University Shanghai Cancer Center, Building 7, No. 270 Dong An Road, Shanghai, 200032 China; Department of Pathology, Fudan University Shanghai Cancer Center, Shanghai, 200032 China; Department of Oncology, Shanghai Medical College, Fudan University, Shanghai, 200032 China

**Keywords:** CDK11^p58^, Metastasis, Integrinβ3, ERα, Tissue array, TaqMan assay

## Abstract

**Background:**

CDK11^p58^, a Ser/Thr kinase that belongs to the cell division cycle 2-like 1 (CDC2L1) subfamily, is associated with cell cycle progression, tumorigenesis and apoptotic signaling. CDK11^p58^ is also involved in the regulation of steroid receptors, such as androgen and estrogen receptors. We previously found that CDK11^p58^ was abnormally expressed in prostate cancer. However, its role in breast cancer remains unclear.

**Methods:**

CDK11^p58^ expression was evaluated by immunohistochemical staining in a tissue array. A Transwell assay was used to detect invasion and metastasis in breast cancer cells. The TaqMan® Metastasis Gene Expression Assay was used to search for potential downstream factors in the CDK11^p58^ signaling pathway. qRT-PCR was used to evaluate mRNA levels, and the dual luciferase array was used to analyze promoter activity. Western blotting was used to detect the protein level.

**Results:**

CDK11^p58^ expression was negatively correlated with node status (*P* = 0.012), relapse status (*P* = 0.002) and metastasis status (*P* = 0.023). Kaplan-Meier survival curves indicated that the disease-free survival (DFS) was significantly poor in breast cancer patients with low CDK11 expression. Interestingly, using the breast cancer cell lines ZR-75-30 and MDA-MB-231, we found that CDK11^p58^ was capable of repressing the migration and invasion of ERα-positive breast cancer cells, but not ERα-negative breast cancer cells, in a kinase-dependent manner. Gene expression assays demonstrated that integrin β3 mRNA was dramatically repressed by CDK11^p58^, and luciferase results confirmed that the integrin β3 promoter was inhibited by CDK11^p58^ through ERα repression. The expression of integrin β3 was highly related to ERα signaling; ERα overexpression stimulated integrin β3 expression, whereas siRNA-mediated knockdown of ERα attenuated integrin β3 expression.

**Conclusions:**

These data indicate that CDK11^p58^ is an anti-metastatic gene in ERα-positive breast cancer and that the regulation of integrin β3 by CDK11^p58^ via the repression of ERα signaling may constitute part of a signaling pathway underlying breast cancer invasion.

## Background

Breast cancer is the most common malignancy among women and represents an important public health issue [1]. It is the major cause of cancer-related death in women, and treatment is particularly difficult in patients with tumor metastasis [2]. Despite recent improvements in survival rates, many patients relapse, and the majority of these patients die from disseminated metastatic disease [3].

The chromosome 1p36 locus is frequently mutated in many types of human tumors, especially in breast tumors. This lesion is associated with progression and lymph node metastasis [4], poor prognosis [5], higher rate of recurrence [6], large tumor size and DNA aneuploidy [7]. Deletion of this chromosome region occurs late in oncogenesis and is correlated with aggressive tumor growth, suggesting that one or more tumor-related genes may reside in this region [8]. However, no specific tumor suppressor gene at 1p36 has yet been identified. Previous studies have identified *CHD5* [9] and *KIF1B* [10] as candidate tumor suppressor genes in this region, and more recently it was reported that disruption of *PER3* function may indicate the likelihood of tumor recurrence in patients with ERα-positive tumors [11]. However, to date, no specific roles for these genes in breast cancer development have been demonstrated.

CDK11^p58^ belongs to the large family of p34cdc2-related kinases [12] and is located on human chromosome 1p36.33, a region frequently mutated in numerous human tumors, including breast cancer. CDK11^p58^ is specifically expressed in the G2/M phase, and expression is closely associated with cell cycle arrest, oncogenesis and apoptosis [13, 14]. Centrosome abnormalities are a common feature of breast cancer [15, 16]. The overexpression of centrosome-related genes has been detected in premalignant lesions, *in situ* breast tumors and over 70% of invasive breast tumors, and is associated with aneuploidy and tumor development [17]. CDK11^p58^ is a centrosome-associated mitotic kinase involved in centrosome maturation and bipolar spindle formation [18] and is required for centriole duplication and Plk4 recruitment to mitotic centrosomes [19]. Furthermore, we found that Thr-370 in CDK11^p58^ is required for its autophosphorylation, dimerization and kinase activity [13].

Previous studies have shown that CDK11^p58^ is involved in the negative regulation of steroid receptors in a kinase activity-dependent manner, including androgen [20], vitamin D [21] and estrogen receptors (ERs) [22]. We previously demonstrated that CDK11^p58^ promotes the ubiquitin/proteasome-mediated degradation of ERα to repress its transcriptional activity [22]. The sex steroid estrogen plays a major role in the development and progression of breast cancer and promotes breast cancer proliferation through a number of established pathways [23]. Estrogen promotes breast cancer cell motility and invasion in ERα-positive breast cancer cells, largely through ERα via FAK and N-WASP [24]. Additionally, the estrogen-ERα complex stimulates the transcriptional activity of MMP-26 and contributes to the survival of ERα-positive breast cancer patients [25]. Because CDK11^p58^ is involved in the negative regulation of the ERα signaling pathway, we speculated whether CDK11^p58^ may function as a tumor suppressor in breast cancer via the inhibition of ERα.

In this study, we demonstrate that CDK11^p58^ inhibited the invasion of ERα-positive breast cancer cells by downregulating integrin β3 expression via ERα signaling. Moreover, the functions of CDK11^p58^ were highly dependent on its kinase activity. These data indicate that CDK11^p58^ plays an important role in the negative regulation of breast cancer invasion.

## Methods

### Patient samples

RNALater-preserved solid tissues, formalin-fixed, paraffin-embedded (FFPE) breast cancer tissues and adjacent normal tissue (ANCT) were obtained from Fudan University Shanghai Cancer Center between 2002 and 2004. The tumors were assessed according to the WHO classification by two academic pathologists. This study was approved by the Ethics Committee of Fudan University Shanghai Cancer Center for Clinical Research. Written informed consent was obtained from all patients.

### Immunohistochemical (IHC) analysis

A total of 250 FFPE blocks of breast cancer tissues and ANCT were collected for tissue microarrays. Two breast cancer tissue cores and two ANCT cores from the same patient’s FFPE blocks were arranged on a recipient paraffin block (with a 1-mm core per specimen). Paraffin sections (3-μm thick) were deparaffinized in xylene and rehydrated in a graded alcohol series, boiled with 10 mmol/L of citrate buffer (pH 6) for 15 min and pre-incubated in blocking solution (10% normal goat sera) for 1 h at room temperature. The steps were performed using the Envision two-step method. The Envision and DAB Color Kit was purchased from Gene Tech Company Limited (Shanghai, China). A rabbit anti-human polyclonal antibody against CDK11 was used at a 1:100 dilution. PBS (phosphate buffered saline) was used as a negative control. The tissue microarray slides were concurrently evaluated by two of the authors. Granular nuclear staining was assessed as positive. The staining index (SI, range 0–9) was considered as the product of the intensity score (0, no staining; 1+, faint/equivocal; 2+, moderate; 3+, strong) and the distribution score (0, no staining; 1+, staining of <10% of cells; 2+, between 10% and 50% of cells; and 3+, >50% of cells). For CDK11 protein in this study, a moderate/strong cytoplasm staining (SI = 3–9) was defined as positive staining, and a weak or negative staining (SI = 0–2) was defined as negative staining.

### Cell culture and transfection

MCF-7, MDA-MB-231, ZR-75-30 and 293 T cells were grown using RPMI1640 supplemented with 10% fetal bovine serum (FBS), 100 units/ml penicillin and 100 μg/ml streptomycin (Cat. 10378-016, Invitrogen, USA) at 37°C and 5% CO_2_. The constructions of CDK11^p58^ mutant plasmids T370A and T370D were mentioned in our previous report [13]. Cell transfection was performed with Lipofectamine 2000 transfection reagent (Cat. 11668-019, Invitrogen) according to the manufacturer’s instructions.

### RNA interference

Small interfering RNAs (siRNA) designed for CDK11 (three siRNAs, siRNA1–3) and ERα (siERα) were ordered from Shanghai GenePharma Co., Ltd. The sequences of the three siRNAs for siCDK11 were as follows: siRNA1, 5′-GAAGCAUGCUAGAGUGAAATT-3′, siRNA2, 5′-GGGAAUGGGAAAGACAGAATT-3′, and siRNA3, 5′-GCAGCAACAUGGACAAGAUTT-3′. The siERα sequence was 5′-GGAGAAUGUUGAAGCACAATT-3′.

### Transwell invasion

Cell invasion was assayed using BD BioCoat Growth Factor Reduced (GFR) Matrigel Invasion Chambers (BD, CA). Transfected ZR-75-30 cells (0.5 ml; 2.5 × 10^4^ cells/ml) were added to the inside of the inserts and incubated for 3 h. After incubation, non-invading cells were removed from the upper surface of the membrane using cotton-tipped swabs. The cells on the lower surface of the membrane were stained with Crystal violet and counted in the central field of triplicate membranes.

### TaqMan® Array human metastasis gene expression assays and quantitative reverse transcription polymerase chain reaction (qRT-PCR)

TaqMan® Array Metastasis 96-Well Plates were obtained from Life Technologies Corporation (California, USA) and contained lyophilized TaqMan® Gene Expression Assays. After converting total RNA to cDNA via reverse transcription, TaqMan® Array Plates and the associated reagents were used to quantitate mRNA expression levels. The results were analyzed using the RQ manager software. Genes altered in the Taqman Array gene expression assays were subsequently verified using a qRT-PCR assay system. Total RNA was extracted using TRIzol reagent (Invitrogen). After converting total RNA to cDNA in a reverse transcription (RT) reaction by Cdna Synthesis Kit (Cat. 6110A, TaKaRa, Dalian, China), qPCR were used to quantitate the mRNA expression levels by SYBR**®** Premix Ex Taq™(Cat. RR420Q, TaKaRa). The cycling conditions were as follows: 95°C for 5 minutes; 40 cycles of 95°C for 15 seconds and 60°C for 32 seconds. GAPDH was used as an endogenous control. Each qRT-PCR cycle was repeated three times to confirm the results.

### Dual luciferase reporter gene assays

Dual luciferase assays were performed as previously described [21]. Luciferase activity was measured using the dual luciferase reporter gene assay (Promega, USA), as previously reported [20], and a SynergyHT Multi-Mode Microplate Reader (BioTek, USA).

### Statistical analysis

The experimental data are expressed as the mean ± standard deviation, and the statistical significance between the different groups was determined using t-tests. The relationship between CDK11 expression and the clinicopathological features of breast cancer patients was analyzed using χ2 and Fisher’s exact tests. All statistical tests were two sided, and *P* values less than 0.05 were considered significant.

## Results

### IHC analysis of CDK11 protein expression

**Figure 1:**
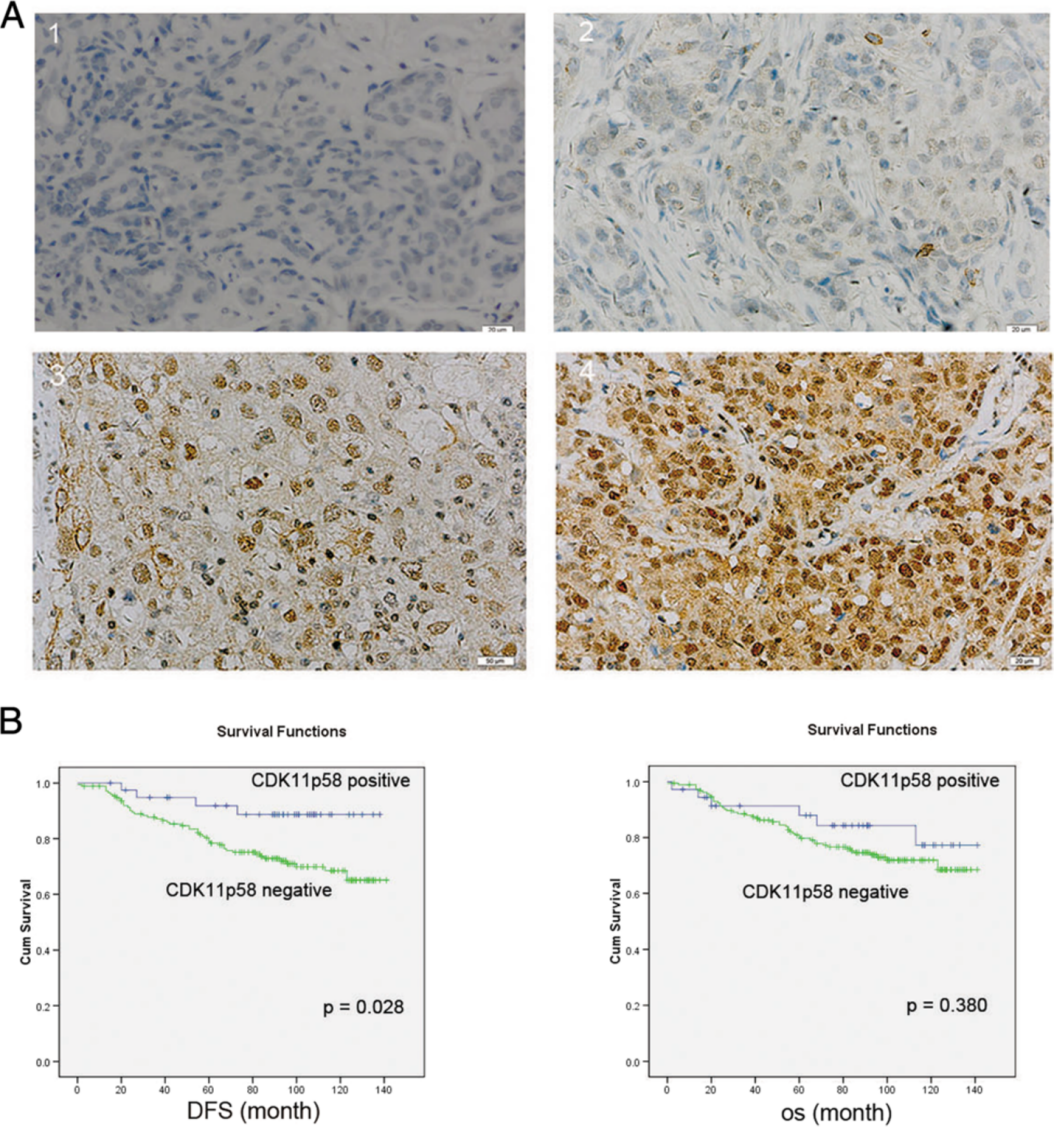
**Immunohistochemistry of CDK11 in tissue microarrays. (A)** CDK11 immunostaining was determined in breast cancer and scored as (1) (2) low expression, (3) (4) high expression. All immunohistochemical photomicrographs are magnified 400×. **(B)** Relationship between CDK11 expression and disease free survival (DFS)/overall survival (OS). *P* values were calculated using the unadjusted log-rank test.

We first evaluated CDK11 protein expression in 250 FFPE breast cancer tissues and ANCTs. CDK11 was clearly expressed in the nucleus in breast cancer tissues, and CDK11 positive expression was found to be both at a higher staining intensity and in a higher proportion of positively stained cells (Figure 1A). Table 1 summarizes the correlation of CDK11 expression with the clinicopathological features of breast cancer patients. CDK11 expression was not correlated with the breast cancer patients’ menopausal status, tumor size, HER-2 status, differentiation or TNM status, but was negatively correlated with age, node status, ER, relapse and metastasis status (*P* < 0.05). The patients with lower CDK11 expression tended to have a higher risk of relapse and metastasis. All patients were followed up for at least 8 years. Disease free survival (DFS) was significantly worse in breast cancer patients with low CDK11 expression (*P* = 0.028; Figure 1B). These results indicate that low CDK11 expression in breast cancer is related to a worse prognosis.

**Table 1:** **Relationship between CDK11** ^**p58**^ **expression and clinicopathological features in breast cancer patients**

Characteristics	CDK11p58				
	Low	High	n	***x*** ^***2***^	***p***
Age(years)				7.024	**0.008**
<50	27(8.1%)	78(31.5%)	105		
≥50	18(7.3%)	125(50.4%)	143		
Menopausal status				0.375	0.540
Pre	29(11.6%)	122(48.8%)	151		
Post	16(6.4%)	83(33.2%)	99		
Tumor size (cm)				0.939	0.333
≦2 cm	18(7.4%)	95(39.1%)	113		
>2 cm	27(11.1%)	103(42.4%)	130		
Node status				6.312	**0.012**
Negative	18(7.2%)	124(49.6%)	142		
Positive	27(10.8%)	81(32.4%)	108		
ER status				4.065	**0.044**
Negative	33(13.2)	117(46.8%)	150		
Positive	12(4.8%)	88(35.2)	100		
HER-2 status				2.823	0.093
Negative	22(8.8%)	128(51.2%)	150		
Positive	23(9.2%)	77(30.8)	100		
Differentiation				1.456	0.228
III	26(13.7%)	142(74.7%)	130		
III	14(7.4%)	49(25.8%)	60		
TNM stage				1.567	0.211
III	23(11%)	129(61.4%)	152		
III	13(6.2%)	45(21.4%)	58		
Relapse				9.323	**0.002**
No	29(11.8%)	179(72.8%)	208		
Yes	13(5.3%)	25(10.2%)	38		
Metastasis				4.803	**0.023**
No	26(10.6%)	159(64.6%)	185		
Yes	16(6.5%)	45(18.3%)	61		

Statistically significant p < 0.05 are indicated in bold. ER, estrogen receptor; PR, progesterone receptor; HER-2, human epidermal growth factor receptor 2.

### CDK11^p58^ inhibits ERα-positive breast cancer metastasis

**Figure 2:**
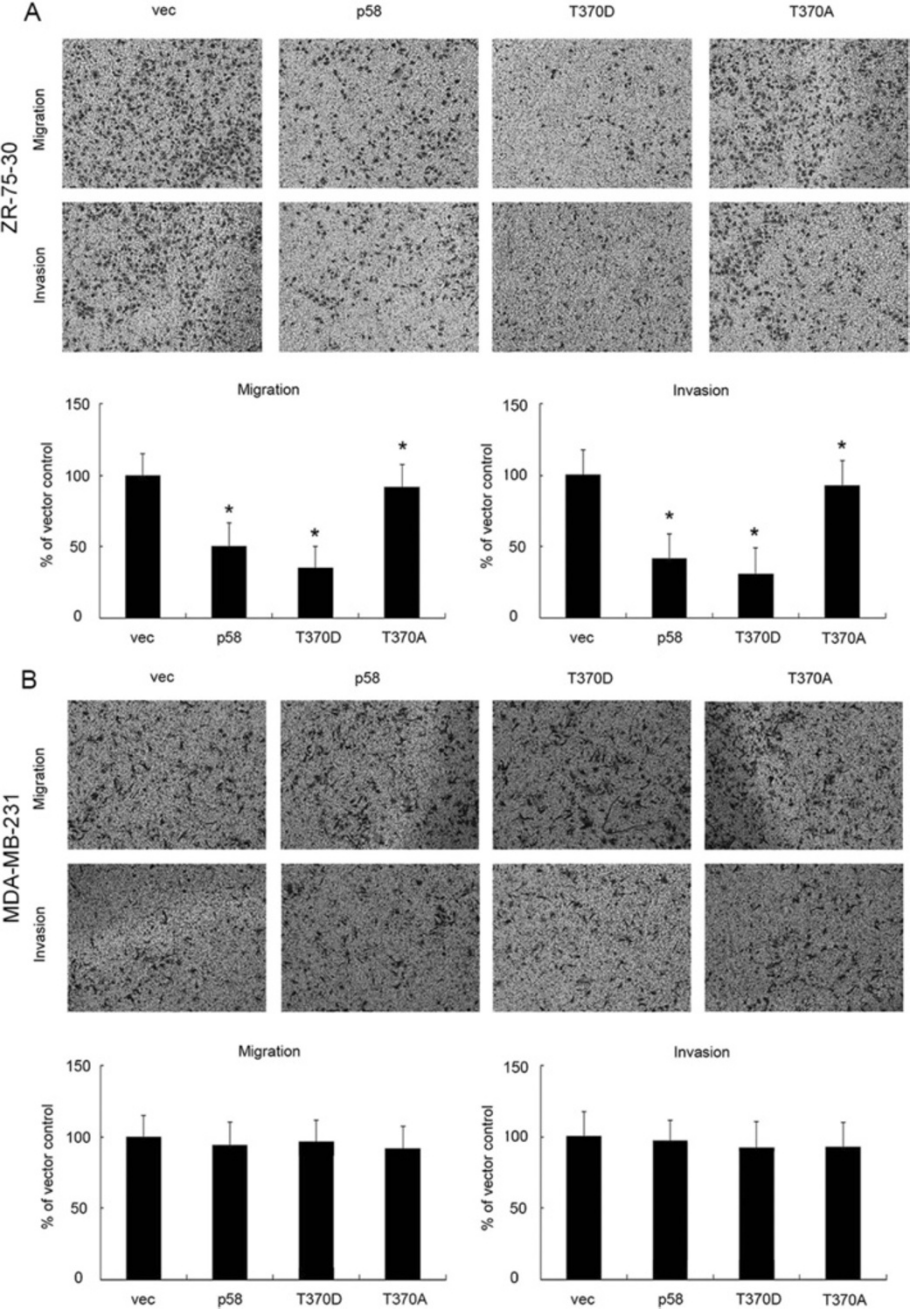
**CDK11** ^**p58**^ **inhibits breast cancer cell metastasis in an ERα-dependent manner. (A)** ZR-75-30 cells were transfected with wild-type CDK11^p58^ (p58), kinase activated mutant (T370D), kinase dead mutant (T370A) or control vectors. After 6 h, Transwell assays were performed as described. Crystal violet staining of invading cells is shown. Data are expressed as the mean ± SEM of the number of invading cells in more than five separate areas. *P < 0.05 versus vector controls (n = 3 experiments). **(B)** MDA-MB-231 cells were transfected with CDK11^p58^ or various mutants. After 6 h, Transwell assays were performed as described. Crystal violet staining of invading cells is shown. Data are expressed as the mean ± SEM of the number of invading cells in more than five separate areas.

As CDK11 expression was negatively correlated with node status, relapse and metastasis status in breast cancer patients, we further investigated the role of CDK11^p58^ in the migration and invasion of breast cancer by Transwell assays. Our previous study showed that the function of CDK11^p58^ was kinase related, and we identified T370D as a constantly activated CDK11^p58^ kinase mutant and T370A as a kinase-dead mutant [13]. The invasion of ZR-75-30 cells transfected with wild-type CDK11^p58^ and the T370D mutant was significantly lower compared with the control cells (*P* < 0.05; Figure 2A). In contrast, the kinase-dead T370A mutant failed to suppress the metastasis of ZR-75-30 cells. Notably, CDK11^p58^ failed to inhibit the migration and invasion of ER-negative MDA-MB-231 breast cancer cells (Figure 2B). Together these data suggest that CDK11^p58^ inhibits ER-positive breast cancer metastasis in a kinase-dependent manner.

### Inhibition of metastasis by CDK11^p58^ is regulated through integrin β3

**Figure 3:**
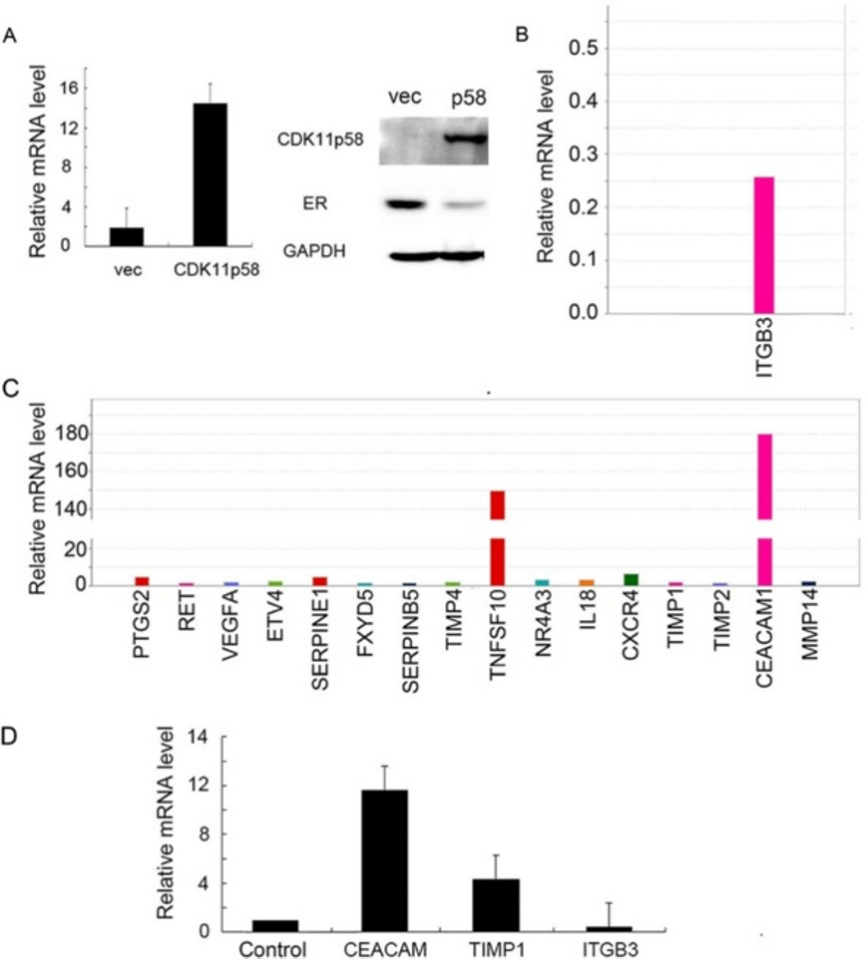
**CDK11** ^**p58**^ **downregulates integrin β3 in ERα-positive breast cancer cells. (A)** Overexpression of CDK11^p58^ in MCF-7 ER-positive breast cancer cells. The expression of CDK11^p58^ was detected by qRT-PCR and western blotting. **(B)** Genes downregulated in the metastasis pathway in the TaqMan Array human metastasis gene expression assay. **(C)** Genes upregulated in the metastasis pathway in the TaqMan Array human metastasis gene expression assay. **(D)** Validation of TaqMan Array human gene expression assays by qRT-PCR.

To investigate the mechanism by which CDK11^p58^ inhibits metastasis, we used TaqMan Array human metastasis expression plates to examine the expression of key genes involved in cancer metastasis. MCF-7 cells were transfected with CDK11^p58^, and the overexpression of CDK11^p58^ mRNA and protein was confirmed by qRT-PCR and western blotting, respectively (Figure 3A). We observed significant changes in the expression of numerous metastasis-related genes in the CDK11^p58^-transfected cells compared with control MCF-7 cells (Figure 3B and C). Notably, integrin β3 was significantly repressed in CDK11^p58^-transfected MCF-7 cells (Figure 3B), and these results were further confirmed by qRT-PCR (Figure 3D). Thus, we speculated that CDK11^p58^ might inhibit the metastasis of breast cancer cells via the repression of integrin β3.

### CDK11^p58^ represses integrin β3 by inhibiting ERα signaling

**Figure 4:**
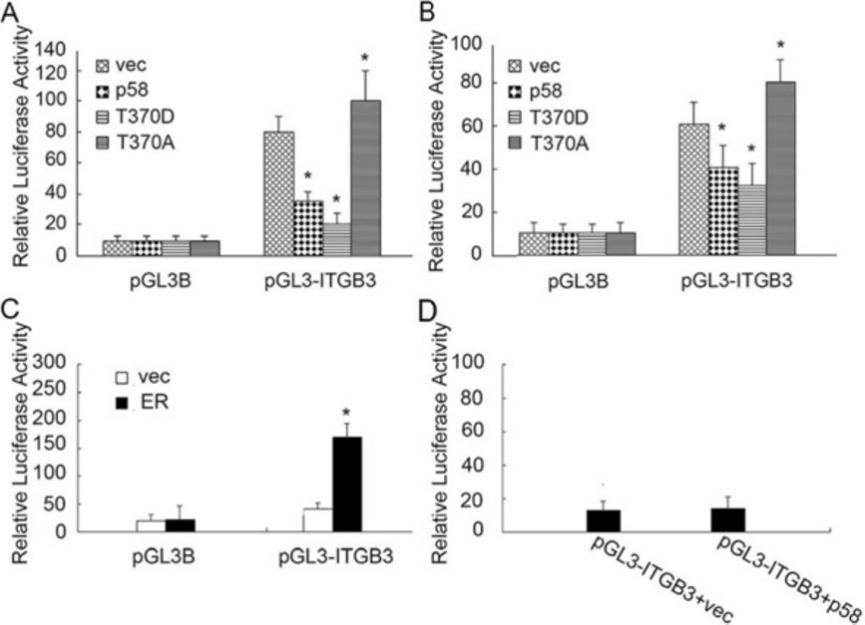
**CDK11** ^**p58**^ **inhibits** ***ITGB3*** **promoter activity in MCF-7 and ZR-75-30 cells. (A and B)** CDK11^p58^ inhibits *ITGB3* promoter activity in a kinase-dependent manner. Dual luciferase assays were performed in ZR-75-30 and MCF-7 cells as described in Methods. *P* < 0.05 vs. controls (n = 3 experiments). **(C)** 293 T cells were transfected with the indicated plasmids, and luciferase assays were performed 48 h later. **(D)** MDA-MB-231 cells were transfected with the indicated plasmids and luciferase assays were performed 48 h later.

As CDK11^p58^ inhibited the mRNA level of integrin β3, we speculated that integrin β3 promoter activity was regulated by CDK11^p58^. We cloned the *ITGB3* promoter (~2.5 kb) spanning -2000 to +50 from the transcriptional start site into the pGL3-Basic (pGL3B) luciferase vector, and transfected the vector into MCF-7 and ZR-75-30 cells for dual luciferase assays (Figure 4A and B). Cotransfection of wild-type or T370D CDK11^p58^ vectors significantly repressed *ITGB3* promoter activity compared with controls, whereas the T370A variant had no effect. These data suggested that CDK11^p58^ inhibited the promoter activity of integrin β3 in a kinase-dependent manner.

We then mapped the promoter region of integrin β3 to identify potential transcription factor binding sites and found three potential estrogen response element (ERE) consensus sites in the -1000 to -500 region. Thus, we speculated that integrin β3 was regulated by ERα. To test this hypothesis, pGL3-ITGB3 and ERα were co-transfected into 293 T cells, and dual luciferase assay results showed that ERα could increase the integrin β3 promoter activity (Figure 4C). We previously demonstrated that CDK11^p58^ represses the transcription activity of ERα. Therefore, we considered that CDK11^p58^ may repress integrin β3 promoter activity through ERα signaling. To further determine whether the inhibition of integrin β3 by CDK11^p58^ is ERα dependent, we performed dual luciferase assays in the MDA-MB-231 ERα-negative cell line. As expected, CDK11^p58^ showed no effect on the integrin β3 promoter in MDA-MB-231 cells (Figure 4D). Together these data suggest that CDK11^p58^ inhibits integrin β3 through ERα signaling.

**Figure 5:**
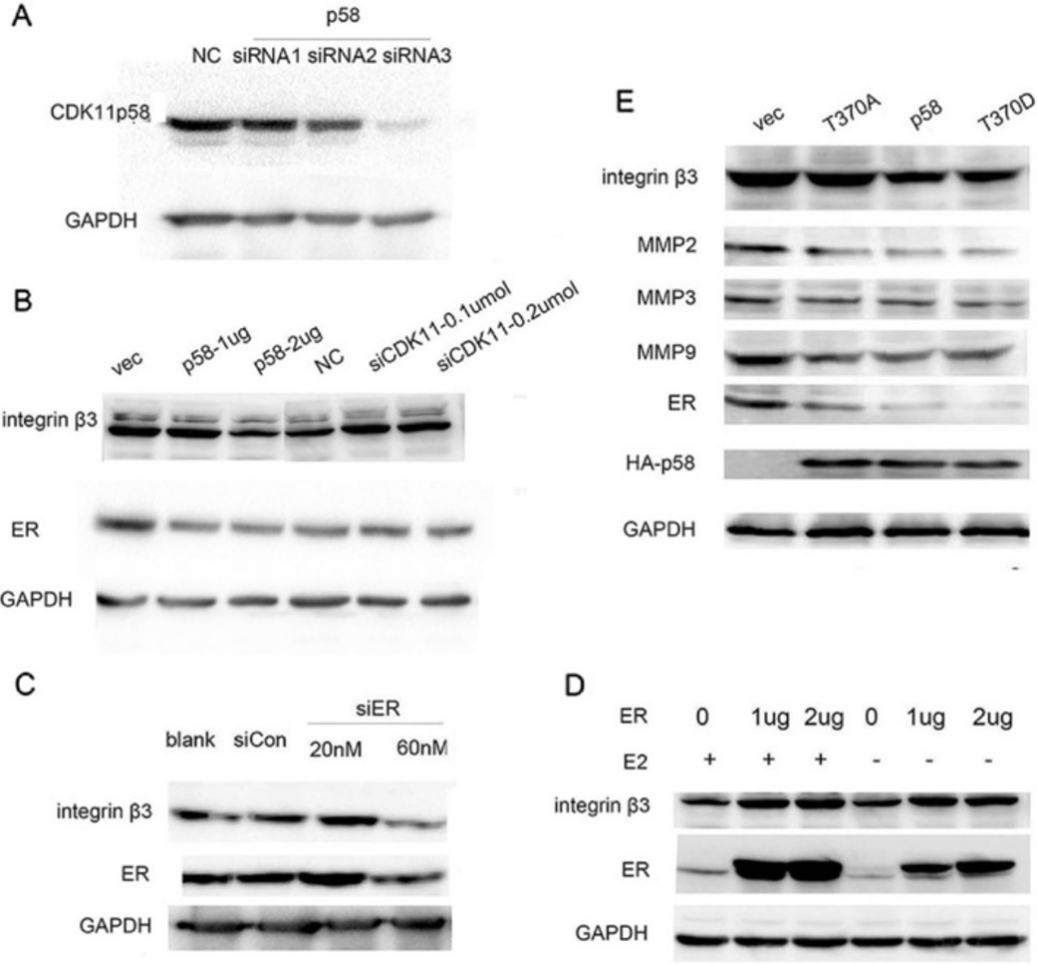
**CDK11** ^**p58**^ **inhibits cell motility and invasion in ERα-positive breast cancer cells through integrin β3. (A)** ZR-75-30 cells were transfected with specific siRNAs targeting CDK11^p58^. After 48 h, the cells were harvested and subject to immunoblotting analysis as indicated. Protein levels were normalized to GAPDH. **(B)** ZR-75-30 cells were transfected with the CDK11^p58^ plasmid or siRNA3 targeting CDK11^p58^ as indicated, and lysates were subjected to an immunoblotting analysis. **(C)** ZR-75-30 cells were transfected with siRNA targeting ERα, and after 48 h, cell lysates were subjected to immunoblotting analysis. **(D)** ZR-75-30 cells were transfected with ERα, and after 36 h, the cells were treated with ethanol or 10 nM E2 for an additional 12 h. The cells were harvested and subjected to immunoblotting analysis as indicated. **(E)** ZR-75-30 cells were transfected with CDK11^p58^ wild-type or mutants. After 48 h, the cells were harvested and the lysates subjected to immunoblotting analysis as indicated.

We then examined this inhibition at the protein level. We first performed CDK11^p58^ knockdown in ZR-75-30 cells using three siRNAs. The third siRNA was the most efficient in knocking down CDK11p58 and was used for subsequent experiments (Figure 5A). Western blotting revealed that the overexpression of CDK11^p58^ attenuated integrin β3 expression, whereas siRNA-mediated knockdown of CDK11^p58^ had no effect to attenuate integrin β3 expression (Figure 5B). Targeted knockdown of ERα by siRNA led to the inhibition of integrin β3 expression, whereas activation of ERα signaling by 17b-estradiol (E2) stimulated integrin β3 expression (Figure 5C and D). The T370D mutant significantly repressed ERα and integrin β3 protein levels, while the T370A mutant failed to repress ERα and integrin β3 protein levels (Figure 5E). Metastasis-related matrix metalloproteinases (MMPs), including MMP2, MMP3 and MMP9, were also repressed by CDK11^p58^ in a kinase-dependent manner. Together these data indicated that CDK11^p58^ represses integrin β3 expression to inhibit the metastasis of breast cancer via the regulation of ERα signaling.

## Discussion

CDK11^p58^ is involved in a variety of important regulatory pathways in eukaryotic cells, including cell cycle control, apoptosis, neuronal physiology, differentiation and transcription [26–28]. CDK11^p58^ is a Ser/Thr kinase, and the majority of its functions are dependent on its kinase activity. In our previous study, we found that CDK11^p58^ inhibited ERα transcriptional activity by promoting its degradation via the ubiquitin-proteasome pathway. In the present study, we demonstrate, for the first time, that CDK11^p58^ expression is involved in the negative regulation of breast cancer invasion in a kinase-dependent manner.

Recent studies have reported the existence of various genomic alterations of 1p36 in ERα-positive breast cancers. CDK11^p58^ is located on chromosome 1p36 and plays a role in the negative regulation of ERα signaling. Approximately 70–75% of breast cancers express ER and/or progesterone receptor. In recent years, we have witnessed tremendous advances in the understanding of ERα biology, revealing a complex process of ERα signaling that includes interactions with other signaling pathways [29]. The ERα signaling pathway plays an important role in the development and progression of breast cancer, and we previously showed that CDK11^p58^ interacts with ERα in breast cancer cells. Estrogens and ERα act as promoters of cell movement in different tissues, including breast tissue [30]. Previous studies showed that ERα induced breast cancer cell migration and invasion via the phosphorylation of FAK and N-WASP [24]. Another report revealed that within the broader range of actions of ERα, rapid extra-nuclear signaling to the actin cytoskeleton through the Gα13/RhoA/ROCK/moesin cascade is relevant for the determination of estrogen-dependent breast cancer cell movement and invasion, which are related to cancer metastasis [31]. Previous studies also demonstrated that the MAPK-integrated signaling crosstalk between Ras and ERα leads to increased breast cancer invasion [32]. Because CDK11^p58^ plays a negative role in the regulation of ERα, we speculated that CDK11^p58^ may participate in the progression of breast cancer via the regulation of the ERα pathway.

First we examined the expression of CDK11 in 250 breast cancer patients and found that high expression of CDK11 indicated a better prognosis, while low expression of CDK11 was related with a worse breast cancer node status, relapse and metastasis. These data suggest that CDK11^p58^ is related to migration and invasion. As expected, CDK11^p58^ inhibited the migration and invasion of ZR-75-30 ERα-positive breast cancer cells in a kinase-dependent manner. To examine the mechanism by which CDK11^p58^ inhibited breast cancer cell invasion, we specifically examined CDK11^p58^-dependent gene expression changes in invasion signaling pathways using TaqMan array human metastasis plates. We observed that the expression of integrin β3 was significantly attenuated in CDK11^p58^-transfected cells. Integrins are cell surface glycoproteins that control cell attachment to the extracellular matrix [33]. Integrins play a role in mammary gland biology, with expression in all cell types within the gland [34], and activate intracellular signaling pathways that control proliferation, differentiation, apoptosis, cell motility, migration and survival [35, 36]. Integrin β3 is expressed on the surface of endothelial cells, smooth muscle cells, monocytes and platelets [37]. Expression is predominantly associated with tumor metastasis and has been reported to increase the metastatic potential of melanoma, breast cancer and lymphoma cells. Interestingly, the opposite effect is observed in ovarian cancer [38].

In this study, we found that integrin β3 promoter activity was significantly repressed by CDK11^p58^ and that this repression was dependent on CDK11^p58^ kinase activity. ERα belongs to the superfamily of nuclear receptors, comprising structurally conserved transcription factors that enhance the transcription of specific genes upon hormone binding [39]. Upon E2 binding, ERα specifically interacts with ERE sequences in target promoters and stimulates the transcription of a variety of E2-responsive genes [40]. As previously published, CDK11^p58^ is capable of repressing the transcriptional activity of ERα and promotes its degradation. In the present study, we further demonstrate that the promoter activity of integrin β3 is stimulated by ERα and that the inhibition of integrin β3 by CDK11^p58^ is dependent on ERα.

Decreased expression of ERα correlated with a decrease in integrin β3 protein. Furthermore, the induction of ERα expression following transfection with ERα plasmids or the activation of ERα signaling with E2 was correlated with the enhanced expression of integrin β3 protein. Overall, CDK11^p58^ and T370D were capable of repressing the expression and transactivation of ERα and ultimately inhibiting the expression of integrin β3, thereby inhibiting the invasion of breast cancer cells. The kinase-dead mutants T370A failed to inhibit the expression of ERα and integrin β3, and were incapable of inhibiting cancer invasion. Taken together, we conclude that CDK11^p58^ may inhibit breast cancer cell invasion via the inhibition of the ERα signaling pathway, leading to the inhibition of integrin β3. Furthermore, metastasis-promoting MMP2, MMP3 and MMP9 proteins were subsequently inhibited. Thus, CDK11^p58^ inhibits the invasion and metastasis of breast cancer via the inhibition of integrin β3.

## Conclusions

Our data show that CDK11^p58^ inhibits the invasion of ERα-positive breast cancer cells by repressing integrin β3. Thus, we demonstrate, for the first time, that CDK11^p58^ is involved in the negative regulation of breast cancer invasion in a kinase activity-dependent manner.
